# Supplementary material for: Tapered photonic switching
Source: Nanophotonics. 2022 Jul 20;11(16):3575–81. doi: 10.1515/nanoph-2022-0200 (PMC11501452; doi:10.1515/nanoph-2022-0200)
Supplement: Supplementary file 1 — Supplementary Material Details [file j_nanoph-2022-0200_suppl.pdf]

Supplemental document: Tapered Photonic Switching

1. Fast Numerical Solver for Continuous Time-Modulation

From Maxwell's Equations we can easily derive

$$\nabla^2 \mathbf{D} = \varepsilon(t)\mu(t)\frac{\partial^2 \mathbf{D}}{\partial t} + \varepsilon(t)\frac{\partial \mu}{\partial t}\frac{\partial \mathbf{D}}{\partial t}. \tag{0.1}$$

Assuming spatially harmonic solutions, we have $\nabla^2 \to -k^2$. Moreover, assuming that the modulation occurs over a finite time-interval, we have $\frac{\partial \varepsilon}{\partial t}_{|t\to\pm\infty} = 0$. Therefore, the solutions before and after the switching has occurred must be of the form

$$\begin{aligned} D(t_a) &= D_a^+ e^{i\omega_a t_a} + D_a^- e^{-i\omega_a t_a} \\ D(t_b) &= D_b^+ e^{i\omega_b t_b} + D_b^- e^{-i\omega_b t_b} \end{aligned}, \tag{0.2}$$

where ω_a is the initial frequency of the wave at $t = t_a$ and $\omega_b = \frac{n_a}{n_b}\omega_a$, $n_a = \sqrt{\varepsilon_a \mu_a}$, and we consider a 1D case without loss of generality. We can therefore solve Eq. (0.1) efficiently as a pair of coupled ODEs for the displacement field $D^{(0)}$ and its time-derivative $D^{(1)}$

$$\frac{\partial}{\partial t}\begin{pmatrix} D^{(0)} \\ D^{(1)} \end{pmatrix} = \begin{pmatrix} 0 & 1 \\ -\frac{k^2}{\varepsilon(t)\mu(t)} & -\varepsilon(t)\frac{\partial \mu}{\partial t} \end{pmatrix}\begin{pmatrix} D^{(0)} \\ D^{(1)} \end{pmatrix} \tag{0.3}$$

via any common ODE solver, subject to the continuity conditions for $D^{(0)}$ and $D^{(1)}$ (note that the latter condition is equivalent to the continuity of the magnetic field B)

$$\begin{aligned} D^{(0)}(t_j) &= e^{i\omega_j t_j} D_j^+ + e^{-i\omega_j t_j} D_j^- \\ D^{(1)}(t_j) &= i\omega_j (e^{i\omega_j t_j} D_j^+ - e^{-i\omega_j t_j} D_j^-) \end{aligned}, \tag{0.4}$$

where $j = a,b$, D_a^+ (D_a^-) is the amplitude of the forward (backward) input wave (i.e. at $t = t_a$) and D_b^+ (D_b^-) is the unknown field amplitude for the total forward (backward) wave at the end of the scattering process (i.e. at $t = t_b$).

2. Fourier transform of the derivative of the sigmoidal permittivity profile

The derivative of the sigmoidal permittivity profile

$$\varepsilon_s = (\varepsilon_1 + \frac{\delta\varepsilon}{2}) + \frac{\delta\varepsilon}{2}\tanh(t/\tau) \tag{0.5}$$

can be readily calculated as

$$\frac{d\varepsilon}{dt} = \frac{\delta\varepsilon}{2\tau}\text{sech}^2\{t/\tau\}, \tag{0.6}$$

and its Fourier transform reads

$$\mathcal{F}[\frac{d\varepsilon}{dt}](\omega) = \sqrt{\frac{\pi}{2}}\frac{\delta\varepsilon}{2}\text{csch}(\frac{\pi\tau\omega}{2})\tau\omega. \tag{0.7}$$

3. Antireflection temporal coating (temporal quarter-wave layer)

A perfect antireflection temporal coating is realized, following [1], by introducing a middle-layer with impedance $Z_{qw} = \sqrt{Z_1 Z_2}$ and duration $T_{qw} = (n_{qw}/n_1)/4$ (where $n_{qw} = \sqrt{\varepsilon_{qw}\mu_{qw}}$ is the refractive index in the middle-layer) between the two regions of time, such that the time-reversals caused by the two switching events have equal amplitude and opposite phase.

4. Klopfenstein taper design

Quoting Eq. (11) in the main text

$$F(t) = \frac{1}{2}\frac{\partial \ln Z_0}{\partial t} = \frac{1}{\pi}\int_{-\infty}^{\infty}\rho(\omega)e^{2i\omega t}d\omega, \tag{0.8}$$

we seek a reflection profile which enables equal-ripple amplitude and tunable bandwidth. This is known from the spatial domain to be the continuous limit of the multi-layer Chebyshev filter [1,2]:

$$\rho(\omega) = \rho_0\frac{\cos[\sqrt{(\omega T)^2 - A^2}]}{\cosh A}e^{i\omega T}, \tag{0.9}$$

where

$$\rho_0 = \frac{Z_2 - Z_1}{Z_2 + Z_1} \simeq \frac{1}{2}\ln\left(\frac{Z_2}{Z_1}\right) \tag{0.10}$$

and the parameter A determines the trade-off between the bandwidth $\omega T > A$ and the maximum ripple size through the relation

$$\rho_{max} = \frac{\rho_0}{\cosh A}. \tag{0.11}$$

Evaluating the Fourier transform of Eq. (0.9) yields the impedance profile [3]

$$\ln Z_0 = \frac{1}{2}\ln(Z_1 Z_2) + \frac{\rho}{\cosh(A)} A^2 \phi(2t/T - 1, A), \tag{0.12}$$

where

$$\phi(t, A) = \int_0^t \frac{I_1(A\sqrt{1-y^2})}{A\sqrt{1-y^2}} dy, \tag{0.13}$$

$I_1(x)$ being the modified Bessel function. Eq. (0.13) can be evaluated efficiently by expanding $I_1(x)$ into a series and integrating term by term [4], which yields

$$\phi(t, A) = \sum_{n=0}^{\infty} a_n b_n \tag{0.14}$$

with the recursively evaluated coefficients

$$\begin{aligned} a_0 &= 1;\ a_n = \frac{A^2}{4n(n+1)} a_{n-1}; \\ b_0 &= \frac{t}{2};\ b_n = \frac{\frac{t}{2}(1-t^2)^n + 2nb_{n-1}}{2n+1}. \end{aligned} \tag{0.15}$$

5. Trade-off between bandwidth and reflection ripple amplitude in Klopfenstein taper

The bandwidth and the constant amplitude of the ripples in the pass-band can be traded off for each other via the parameter A, which also determines the maximum ripple amplitude R_{max} through the relation (0.11). Note that the small-reflection assumption results in a discontinuity of the Klopfenstein impedance profile near the edges of the taper which is negligible for smaller bandwidths, but increases as A is reduced, becoming dominant once the bandwidth of the taper is pushed towards lower frequencies. The taper gradually becomes equivalent to the quarter-wave layer once its bandwidth is pushed towards the first minimum of the latter, as shown in Figure S1 for four values of A.

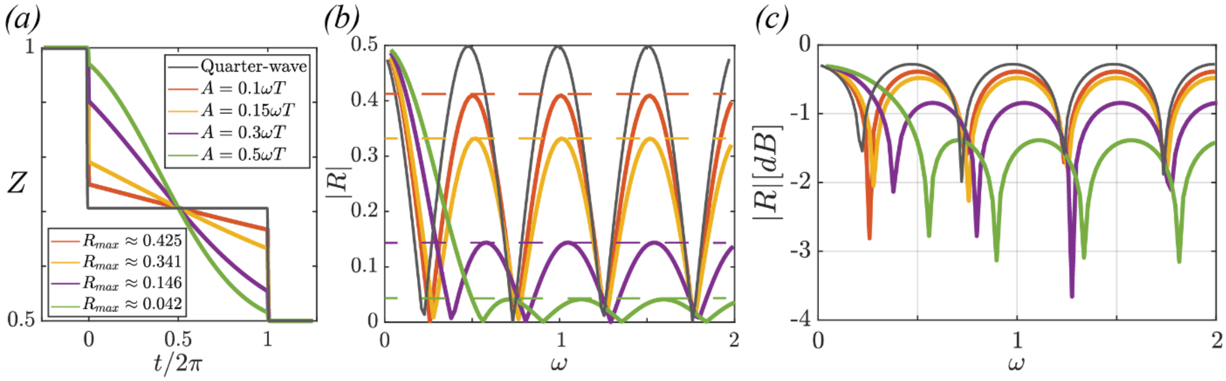

Figure S1: Dependence of the (left) Klopfenstein taper profile and (center, right) response on the bandwidth (ripple amplitude) parameter A (R_{max}) in linear (center panel) and logarithmic (right panel) scale.

6. Comparison between common tapering profiles

Common impedance tapers include the exponential and the triangular filters, as well as the optimal Klopfenstein one [1]. Figure S1 compares the response of the different filters, where the Klopfenstein filter was generated using $A / \omega_1 T = 0.75$. Note how the Klopfenstein taper provides a trade-off between bandwidth (which can be tuned via the parameter A) and maximum ripple amplitude in the pass band (which is ≈ 0.0062 in this case).

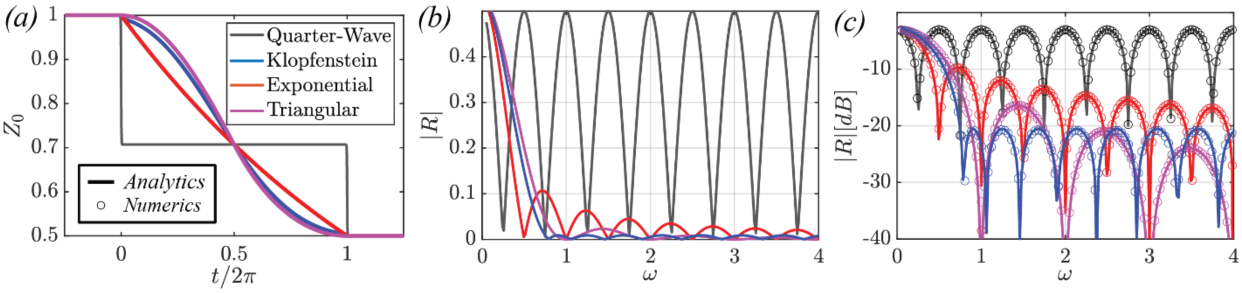

Figure S2: Comparison of temporal quarter-wave layer (grey), and Klopfenstein (blue), exponential (red) and triangular (magenta) tapers.

7. Non-isorefractive tapers

Although the analytical formalism for the design of temporal tapers is quantitatively more accurate in the isorefractive case, the key phenomenology presented is not exclusive to this scenario. Fig. S3 shows the performance of the different tapers for the case where the change in impedance is caused by a change in permittivity alone $\varepsilon : 1 \rightarrow 4$, such that the taper is no longer isorefractive. As for the previous scenario, in fact, the exponential taper offers a broader bandwidth, the triangular one a lower ripple amplitude (although in this case its performance is worse than the Klopfenstein filter over a broader frequency band and improves at higher frequencies) while the Klopfenstein offers an optimal tradeoff. Furthermore, note how the constant-ripple feature of the Klopfenstein taper is unaffected in the non-isorefractive case.

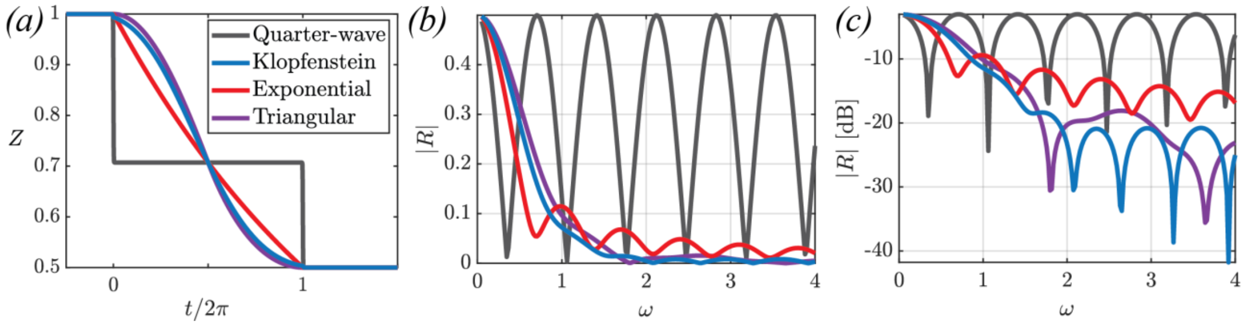

Figure S3: Comparison of different non-isorefractive temporal tapers.

References:

[1] V. Pacheco-Peña and N. Engheta, "Antireflection temporal coatings," *Optica,* vol. 7, p. 323–331, 2020.

[2] R. E. Collin, "Theory and design of wide-band multisection quarter-wave transformers," *Proceedings of the IRE,* vol. 43, p. 179–185, 1955.

[3] T. T. Taylor, Dolph Arrays of Many Elements, Hughes Aircraft Co., 1953.

[4] R. W. Klopfenstein, "A transmission line taper of improved design," *Proceedings of the IRE,* vol. 44, p. 31–35, 1956.

[5] M. A. Grossberg, "Extremely rapid computation of the Klopfenstein impedance taper," *Proceedings of the IEEE,* vol. 56, p. 1629–1630, 1968.
